# Air Pollution, Temperature, and Demographic Vulnerability as Determinants of Cause-Specific Mortality in Portugal

**DOI:** 10.3390/jox16050172

**Published:** 2026-09-12

**Authors:** João Simões, Alexandra Bernardo, Luísa Lima Gonçalves, José Brito

**Affiliations:** 1 Egas Moniz School of Health & Science, Campus Universitário, Quinta da Granja, Monte de Caparica, 2829-511 Caparica, Portugal; abernardo@egasmoniz.edu.pt (A.B.); lgoncalves@egasmoniz.edu.pt (L.L.G.); jbrito@egasmoniz.edu.pt (J.B.); 2 Egas Moniz Center for Interdisciplinary Research (CiiEM), Egas Moniz School of Health & Science, Campus Universitário, Quinta da Granja, Monte de Caparica, 2829-511 Caparica, Portugal

**Keywords:** air pollution, cause-specific mortality, NO_2_, PM_2.5_, temperature, demographic heterogeneity

## Abstract

Air pollution and temperature are important environmental risk factors for mortality, but variation in risks across causes of death, population groups, and co-exposure conditions remains incompletely characterised. This study used nationwide monthly data from Portugal for the period 2010–2022 to estimate cause-specific mortality risks associated with nitrogen dioxide (NO_2_), fine particulate matter (PM_2.5_), and temperature, while evaluating sex–age interaction structures and selected co-exposure patterns. Mortality from malignant tumours, circulatory diseases, and respiratory diseases was modelled using Generalised Estimating Equations to account for temporal correlation and interaction effects. Pollutant exposures were defined as concentrations exceeding the 2021 World Health Organization air quality guideline values. Associations varied by cause of death. Respiratory mortality showed the strongest environmental association, with a 1.4% increase in risk per 1 μg/m^3^ increase in NO_2_ above the guideline value (RR 1.014, 95% CI 1.009–1.019), and the highest predicted risk was observed among males aged ≥65 years (RR 142.610, 95% CI 142.200–143.020). Circulatory mortality was also associated with NO_2_ (RR 1.005, 95% CI 1.001–1.008) and showed a submultiplicative sex–age interaction (RR 0.344, 95% CI 0.343–0.344), indicating attenuation of sex differences at older ages. Malignant tumour mortality showed weaker environmental associations but a greater-than-multiplicative sex–age interaction (RR 1.117) and attenuation of the NO_2_ association at higher PM_2.5_ levels, although the pollutant-related findings were less robust in sensitivity analyses. These findings indicate that mortality risks are not uniform across causes of death or population groups, supporting the value of locally derived, demographically stratified estimates for environmental health assessment and targeted public health strategies.

## 1. Introduction

Air pollution is widely recognised as one of the leading environmental risks to public health worldwide, remaining a persistent challenge despite decades of regulatory measures and technological progress [1]. In 2023, air pollution accounted for an estimated 7.9 million deaths globally, representing approximately one in eight deaths worldwide and ranking as the second leading risk factor for premature mortality after high blood pressure [2]. This burden is largely driven by non-communicable diseases, highlighting the chronic and pervasive nature of population exposure.

Against this global evidence base, a substantial body of epidemiological evidence links exposure to specific air pollutants with increased risks of all-cause and cause-specific mortality. PM_2.5_ has been consistently associated with increased risks of ischaemic heart disease, stroke, lung cancer, and chronic obstructive pulmonary disease (COPD), owing to its capacity to penetrate deeply into the respiratory tract and induce systemic effects [3].

Nitrogen dioxide (NO_2_), emitted predominantly through high-temperature combustion processes, particularly road traffic, is consistently associated with respiratory morbidity and mortality and is commonly used as an indicator of the traffic-related pollution mixture in urban environments, where it acts both as a directly harmful pollutant and as a proxy for complex traffic-related mixtures that include particulate matter and other co-pollutants [4,5]. From a health perspective, NO_2_ has been shown to trigger airway inflammation and oxidative stress [4] and is consistently associated with asthma exacerbations, COPD, respiratory hospital admissions, and increased mortality [5]. At the same time, NO_2_ correlates strongly with other traffic-related pollutants, and epidemiological associations may therefore reflect a combination of direct NO_2_ toxicity and broader mixture-related effects [4].

In addition, the 2021 World Health Organization (WHO) air quality guidelines (AQG) established substantially lower recommended exposure levels for NO_2_ and PM_2.5_ [6], reflecting growing evidence of adverse health effects even at relatively low concentrations. In this context, defining exposure relative to these guideline values provides a policy-relevant framework for assessing health risks and identifying excess exposure above recommended thresholds, which underpins the construction of exposure metrics used in this study.

Temperature constitutes an important environmental stressor and is associated with increased mortality under both heat and cold extremes, including in the Portuguese context [7,8]. In addition to its direct health impacts, temperature influences atmospheric processes and exposure conditions, shaping the formation, dispersion, and co-occurrence of air pollutants [9,10]. These dynamics underscore the importance of considering temperature not only as an independent exposure but also as a contextual factor that may modify the health effects of air pollution [11].

Despite major advances in burden-of-disease assessment, important gaps remain in understanding how environmental exposures interact across different population and exposure contexts [12,13]. Frameworks such as the Global Burden of Disease (GBD) study and WHO AirQ+ provide robust and policy-relevant estimates of attributable mortality and have substantially advanced the quantification of the global health burden associated with air pollution [13,14]. Nevertheless, incomplete knowledge remains regarding the role of multipollutant exposure, environmental interactions, and contextual heterogeneity in shaping mortality risk under real-world exposure conditions [15].

In particular, growing evidence suggests that mortality risks associated with air pollution may vary according to pollutant mixtures, meteorological conditions, demographic structure, and co-exposure patterns [16,17]. While conventional burden-of-disease approaches necessarily rely on standardised exposure–response assumptions for large-scale population assessment [13,16,17], complementary analytical approaches are often needed to explore complex interaction structures and contextual variability across exposure conditions, causes of death, and population subgroups [18]. More integrated modelling approaches that jointly consider demographic heterogeneity, temperature, and multipollutant exposure may therefore provide additional insight into how mortality risks vary across exposure conditions and population groups, particularly in ageing populations that may present increased vulnerability to environmental stressors [13,17,18].

In Portugal, evidence on mid to short-term mortality effects of air pollution remains limited, particularly with respect to analyses that jointly consider multiple pollutants, temperature, and population heterogeneity [9,19]. Existing studies have predominantly focused on single-pollutant models or specific regions, with less attention given to integrated national analyses that simultaneously account for co-exposure and demographic variability. Moreover, evidence remains scarce on how these associations and mortality patterns vary across demographic groups, particularly by age and sex, despite their known importance as determinants of vulnerability [20]. Furthermore, few studies have systematically evaluated interaction structures between pollutants, between pollutants and temperature, and between demographic factors within a unified modelling framework.

Building on previous national assessments of attributable mortality [21] and analyses of seasonal and temporal patterns [22], the present study conducts a population-based analysis of mortality in Portugal using data aggregated at the monthly level, capturing medium-term temporal variation in exposure and mortality patterns. Mortality from malignant tumours (MNs), circulatory system diseases (CVD), and respiratory system diseases (RSD) is modelled as a function of concentrations of NO_2_ and PM_2.5_, together with mean temperature (T_mean_). Generalised Estimating Equations (GEE) with a negative binomial distribution and population offset are used to account for overdispersion and temporal correlation in the data.

A structured modelling framework was applied to quantify associations between air pollution, temperature, and cause-specific mortality, and to evaluate whether selected interaction terms improved model fit beyond main effects. Specifically, the analysis assessed interactions between pollutants, between pollutants and temperature, and between sex and age group, while estimating mortality risks across demographic groups after accounting for environmental exposure conditions. By combining cause-specific mortality, demographic stratification, multipollutant exposure, temperature, and exceedance metrics based on WHO guideline values, this study aims to provide locally derived and policy-relevant evidence on mortality risk in Portugal. In doing so, it contributes complementary evidence on co-exposure patterns, sex–age differences in mortality risk, and cause-specific susceptibility, supporting more integrated environmental health assessment frameworks and targeted public health interventions in ageing populations increasingly exposed to environmental risks.

## 2. Materials and Methods

### 2.1. Dataset

This study adopted a nationwide population-based study design using mortality, population, and environmental data for Portugal, covering all 25 NUTS III regions according to the NUTS 2013 classification. The original dataset comprised daily observations from 1 January 2010 to 31 December 2022, which were subsequently aggregated at the monthly level to capture medium-term temporal variation while reducing short-term variability. Data were structured as repeated observations over time within sex and age strata, integrating mortality, population, and environmental information to evaluate main effects and higher-order interactions between air pollution, temperature, sex, and age group.

#### 2.1.1. Health and Population Data Sources

Daily mortality data were obtained from Statistics Portugal (Instituto Nacional de Estatística, INE) for the period 2010–2022, following a formal data request under restricted access conditions. Mortality counts corresponded to the number of daily deaths by cause of death, sex, age group, and place of residence, aggregated at the NUTS III regional level, and subsequently aggregated at the monthly level for analysis.

The analysis focused on three major cause-of-death groups selected for their public health relevance and established sensitivity to environmental exposures: malignant tumours (MNs; corresponding to malignant neoplasms, ICD-10 codes C00–C97), diseases of the circulatory system (CVD; ICD-10 I00–I99), and diseases of the respiratory system (RSD; ICD-10 J00–J99). Mortality data were stratified by sex (male, female) and age group (0–64 years and ≥65 years).

Population data used as denominators were also obtained from Statistics Portugal and were based on official census data and intercensal population estimates. Population counts were stratified by sex and age group (0–64, and ≥65 years). Annual population estimates were used as population denominators for each corresponding calendar year and incorporated in the models as an offset representing the population at risk, thereby allowing the estimation of mortality rates rather than crude counts.

#### 2.1.2. Air Pollution Data

Air pollution data were obtained from the Portuguese Environment Agency (Agência Portuguesa do Ambiente, APA) through the Online Air Quality Database (QualAr) for the period 1 January 2010 to 31 December 2022. Hourly concentrations of nitrogen dioxide (NO_2_), particulate matter with aerodynamic diameter ≤ 10 μm (PM_10_), and particulate matter with aerodynamic diameter ≤ 2.5 μm (PM_2.5_) were collected from fixed monitoring stations distributed across Portugal.

Only monitoring stations with at least 75% of valid hourly observations, as validated and approved by APA, were included. This threshold is consistent with EEA air-quality assessment practice, whereby a minimum data coverage of 75% is used for assessment purposes to retain a larger number of monitoring stations without a significant increase in monitoring uncertainty [23,24]. For each pollutant, hourly measurements were aggregated into daily mean concentrations at the station level. Stations classified as urban, suburban, rural, and traffic were included to represent the range of ambient pollution conditions recorded across the Portuguese monitoring network. Station-period observations not meeting the 75% data-coverage criterion were excluded from the corresponding estimates, ensuring a consistent data-completeness criterion across monitoring locations and periods. The resulting national exposure metric reflects the mean concentration recorded across the monitoring network for each period and is intended to capture temporal variation in national ambient pollution levels. It should therefore be interpreted as a national-level temporal exposure indicator rather than a population-weighted or spatially resolved estimate.

National air pollution indicators were first computed as the arithmetic mean of daily concentrations across all monitoring stations reporting valid data for each pollutant and were subsequently aggregated at the monthly level, using the arithmetic mean of daily values, to match the temporal resolution of the epidemiological analysis, thereby aligning exposure assessment with the outcome aggregation framework. The spatial distribution and characteristics of the monitoring stations are publicly available through the QualAr database [25].

For fine particulate matter (PM_2.5_), measured concentrations were used whenever available. For monitoring stations that reported PM_10_ but did not provide PM_2.5_ measurements, PM_2.5_ concentrations were approximated as 45% of the corresponding PM_10_ values. This conversion was applied only when direct PM_2.5_ measurements were unavailable and is consistent with approaches adopted in earlier epidemiological studies [19,21]. The factor of 0.45 was empirically derived as the weighted mean of observed PM_2.5_-to-PM_10_ ratios at Portuguese monitoring stations with valid measurements for both pollutants and closely agrees with the mean conversion factor of 0.46 (range: 0.32–0.63) reported for Portugal in the WHO Ambient Air Quality Database for 2016 [26]. The annual numbers and proportions of monitoring stations contributing directly measured PM_2.5_ concentrations and PM_2.5_ concentrations estimated from PM_10_ are provided in Supplementary Table S1.

The air quality monitoring network operates in accordance with the European Air Quality Directive (2008/50/EC).

#### 2.1.3. Temperature Data

Temperature data were obtained from the Portuguese Institute for Sea and Atmosphere (Instituto Português do Mar e da Atmosfera, IPMA), using in situ observations of 2-metre air temperature (Tair2m) recorded at meteorological stations across Portugal. The dataset covered the period from 1 January 2010 to 31 December 2022 and included daily minimum, mean, and maximum air temperatures.

National daily temperature indicators were calculated as the arithmetic mean across stations with valid observations and subsequently aggregated into monthly averages to match the temporal resolution of the epidemiological analysis.

#### 2.1.4. Key Variables for Analysis

The analyses were based on a set of demographic, environmental, temporal, and health outcome variables defined to evaluate main effects and higher-order interactions between air pollution, temperature, sex, age group, and calendar month.

Air pollution exposures were defined as monthly mean concentrations of nitrogen dioxide (NO_2__mean) and particulate matter (PM_2.5__mean), as described above, and modelled as continuous variables. In addition, excess pollutant concentrations were defined relative to the 2021 World Health Organization (WHO) air quality guideline (AQG) values, such that:

NO_2__Excess = max(NO_2__mean − 10, 0)

PM_2.5__Excess = max(PM_2.5__mean − 5, 0)

where concentrations are expressed in µg/m^3^. These variables represent the amount by which observed concentrations exceeded the WHO guideline thresholds.

Temperature exposure was represented by monthly mean temperature (T_mean_), also included as a continuous variable, consistent with its use as an integrated indicator of overall thermal conditions in epidemiological studies.

Sex [female (reference), male] and age group [0–64 years (reference), ≥65 years] were included as categorical variables to assess potential differences in susceptibility across demographic strata.

The health outcomes consisted of monthly counts of cause-specific mortality, including malignant tumours (MNs), diseases of the circulatory system (CVD), and diseases of the respiratory system (RSD), which were analysed separately.

To account for differences in the population at risk, the natural logarithm of the population size was included in all models as an offset term, allowing the estimation of relative risks within a generalised linear modelling framework.

### 2.2. Statistical Methods

Mortality counts were analysed using Generalised Estimating Equations (GEE) with a log link function and a negative binomial distribution to account for overdispersion. Models were estimated using quasi-likelihood methods as implemented in IBM SPSS Statistics (version 30.0, IBM Corp., Armonk, NY, USA).

To model mortality rates rather than crude counts, the natural logarithm of the population at risk was included as an offset term. The general model specification was:

$$\log\left(\mu_{t,s}\right)=\beta_{0}+\sum_{i}\beta_{i}Z_{i,t,s}+\sum_{j<l}\beta_{jl}\left(Z_{j,t,s}\times Z_{l,t,s}\right)+\log\left({Population}_{t,s}\right)$$

where $\mu_{t,s}$ denotes the expected mortality count in month t and demographic stratum s, $Z_{i,t,s}$ represents the set of fixed-effect covariates, including calendar month, environmental exposures, and demographic variables. Interaction terms $\left(Z_{j,t,s}\times Z_{l,t,s}\right)$ were included to evaluate effect modification between environmental exposures and demographic variables.

A negative binomial variance structure (NB2) was specified to accommodate overdispersion. To account for within-series correlation, models were fitted using alternative working correlation structures, including independent and first-order autoregressive [AR(1)] structures. The optimal correlation structure was selected using the quasi-likelihood under the independence model criterion (QIC), with lower values indicating improved model fit.

Regression coefficients were estimated on the logarithmic scale and exponentiated to obtain relative risks (RRs) and corresponding 95% Wald confidence intervals. For models including interaction terms, stratum-specific RRs were derived from estimated marginal means and linear contrasts on the log scale and subsequently exponentiated. Standard errors were obtained from the variance–covariance matrix of the model estimates.

### 2.3. Model Selection and Diagnostics

Model selection followed a pre-specified hierarchical strategy to evaluate the contribution of interaction terms while preserving interpretability and model stability.

A baseline model (M0) including calendar month, sex, age group, NO_2__Excess, PM_2.5__Excess, and T_mean_ was specified for all analyses. A set of nested models (M0–M4) was then defined to evaluate selected interaction structures involving demographic variables and environmental exposures. Model M1 incorporated the interaction between sex and age group to assess demographic effect modification. Model M2 additionally included the interaction between NO_2__Excess and PM_2.5__Excess. Models M3 and M4 evaluated interactions between pollutants and temperature (NO_2__Excess × T_mean_ and PM_2.5__Excess × T_mean_, respectively).

Interaction terms were introduced according to hierarchical modelling principles, such that higher-order interactions were only considered when their lower-order components were retained.

Each candidate model was fitted using alternative working correlation structures (independent and AR(1)). Among models achieving convergence and stable parameter estimates, QIC was pre-specified as the primary criterion for final model selection. QIC was prioritised because the comparison involved both alternative mean-model specifications and alternative working correlation structures and provides an established GEE-specific criterion applicable to both model and working correlation structure selection [27]. The specification yielding the lowest QIC was selected. QICc was calculated and reported as complementary information but was not used to override the QIC-based ranking. No fixed numerical ΔQIC or ΔQICc threshold was applied. This decision rule was applied consistently across all three mortality outcomes.

As a sensitivity analysis, the selected model for each mortality outcome was re-estimated including a binary indicator for the COVID-19 pandemic period (2020–2022), with 2010–2019 as the reference period. This analysis was performed to assess whether the main environmental and demographic associations were robust to the broad temporal changes in mortality patterns and environmental exposures associated with the pandemic period.

## 3. Results

### 3.1. Descriptive Statistics

Table 1 summarises descriptive statistics of monthly mortality counts, air pollutant concentrations, and mean temperature in Portugal over the study period 1 January 2010 to 31 December 2022. The analytical dataset consisted of monthly observations derived from the aggregation of daily data. Mortality counts were stratified by cause of death, sex, and age group (0–64 and ≥65 years), while environmental variables, including nitrogen dioxide (NO_2_), fine particulate matter (PM_2.5_), and mean temperature (T_mean_), were calculated as monthly averages based on daily measurements. Descriptive statistics are presented as mean, standard deviation, and selected percentiles (P_25_, median, P_75_).

Across all causes of death, mortality was substantially higher among individuals aged ≥65 years compared with those aged 0–64 years. Circulatory system diseases (CVD) represented the largest share of mortality, particularly in older age groups, followed by malignant tumours (MNs) and respiratory system diseases (RSD). This age gradient was especially pronounced for circulatory mortality, with notably higher counts observed among older individuals, particularly females.

Differences by sex were also evident. Mortality was generally higher among males than females in younger age groups, particularly for malignant tumours and circulatory diseases. However, among older individuals, sex differences were less pronounced for some outcomes, especially for respiratory mortality.

Environmental exposures showed moderate variability over the study period. Short-term exceedances of World Health Organization (WHO) 24-h guideline values were observed, with NO_2_ concentrations exceeding 25 µg/m^3^ on 18.7% of days and PM_2.5_ exceeding 15 µg/m^3^ on 9.0% of days. Mean temperature exhibited clear seasonal variability, reflecting the climatic conditions of Portugal.

Overall, the descriptive analysis highlights marked differences in mortality across age groups and causes of death, as well as recurring exceedances of air pollutants above recommended thresholds, providing important context for the subsequent modelling analyses.

### 3.2. Model Selection

Model selection results are summarised in Table 2. Distinct patterns were observed across outcomes. For malignant tumour mortality, the model including both demographic and pollutant interaction terms (M2) provided the best fit, indicating a modest interaction between NO_2_ and PM_2.5_. For both circulatory and respiratory mortality, the model including the interaction between sex and age group (M1) was selected, highlighting the importance of demographic effect modification. In all cases, the AR(1) correlation structure improved model fit compared with the independent structure. Detailed results of model selection are provided in Supplementary Tables S2–S4.

### 3.3. Model Results

#### 3.3.1. Malignant Tumours Mortality (MNs)

For malignant tumour mortality, a clear seasonal pattern was observed, with most months showing lower mortality compared to December. The largest reductions were observed in February (RR = 0.899; 95% CI: 0.888–0.910) and April (RR = 0.925; 95% CI: 0.913–0.938), while smaller but significant decreases were also found in March, May, June, September, and November. No significant differences were observed for July, August, or October.

Detailed parameter estimates are provided in Table S5 (Supplementary Material), and key model estimates are presented in Table 3.

Substantial demographic differences were observed. Mortality was markedly higher among individuals aged ≥65 years compared to those aged 0–64 (RR = 10.451; 95% CI: 10.441–10.461; *p* < 0.001), and higher among males compared to females (RR = 1.728; 95% CI: 1.727–1.729; *p* < 0.001). A significant interaction between sex and age group (RR = 1.1169; 95% CI: 1.1167–1.1171; *p* < 0.001) indicated that the combined effect of male sex and older age was greater than multiplicative, suggesting an amplification of risk in older males beyond the sum of individual effects. Differences in baseline mortality across demographic groups are further detailed in Table S6 (Supplementary Material).

Regarding environmental exposures, a small but statistically significant association was observed for NO_2_ excess (RR = 1.003 per 1 µg/m^3^; 95% CI: 1.001–1.006; *p* = 0.019), whereas no independent association was found for PM_2.5_ excess or mean temperature.

**Figure 1:**
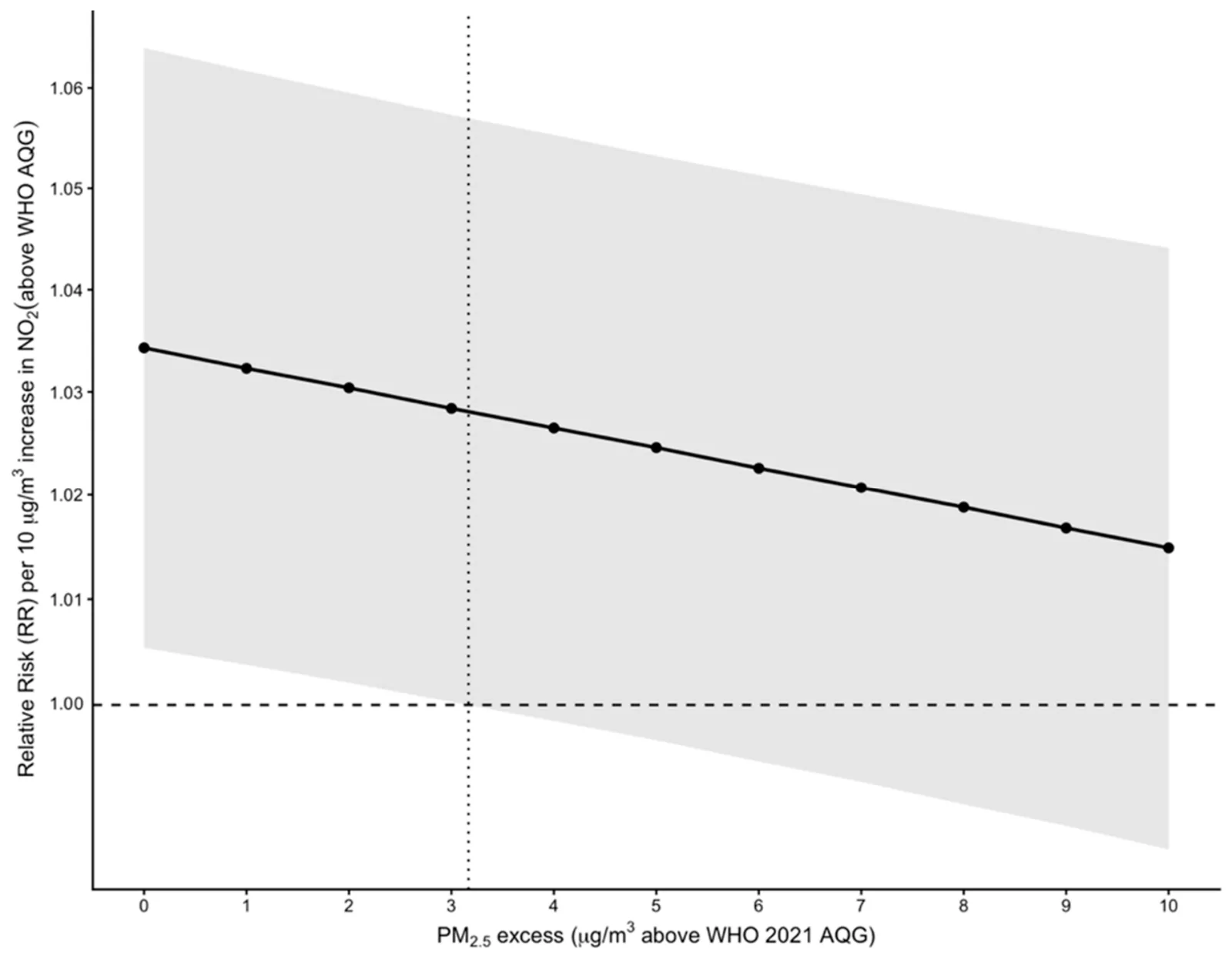
Effect modification of the association between NO_2_ and malignant tumour mortality by PM_2.5_ levels.

The interaction between NO_2_ and PM_2.5_ is illustrated in Figure 1. The conditional effects of NO_2_ varied according to PM_2.5_ levels. At low PM_2.5_ concentrations, NO_2_ was positively associated with mortality, with a 10 µg/m^3^ increase corresponding to a 3.4% increase in risk when PM_2.5_ excess was zero (RR = 1.034; 95% CI: 1.005–1.064). This association progressively weakened with increasing PM_2.5_, with the estimated relative risk approaching the null. Statistical significance was lost at approximately 3–4 µg/m^3^ of PM_2.5_ excess, beyond which confidence intervals included the null value. Detailed conditional estimates are provided in Table S7 (Supplementary Material), and the interaction pattern across increasing levels of NO_2_ excess is further illustrated in Figure S1 (Supplementary Material).

#### 3.3.2. Circulatory System Mortality (CVD)

Circulatory system mortality also exhibited a marked seasonal pattern, characterised by higher mortality in January (RR = 1.088; 95% CI: 1.060–1.116) and lower mortality in subsequent months compared to December. The largest reductions were observed in September (RR = 0.811; 95% CI: 0.730–0.901), October (RR = 0.845; 95% CI: 0.785–0.910), and November (RR = 0.865; 95% CI: 0.841–0.889). No significant differences were observed for March, July, or August.

Key model estimates for circulatory system mortality are presented in Table 4.

Substantial differences by age group and sex were observed, with markedly higher mortality among older individuals and males. However, a significant interaction between sex and age group (RR = 0.344; 95% CI: 0.343–0.344; *p* < 0.001) indicated that the combined effect of male sex and older age was less than multiplicative. In other words, although both factors were independently associated with increased mortality, their joint effect was attenuated when occurring together. This pattern is further illustrated in Table S9 (Supplementary Material), where the excess risk associated with male sex is markedly reduced among older individuals.

Regarding environmental exposures, NO_2_ excess was positively associated with circulatory mortality (RR = 1.005 per 1 µg/m^3^; 95% CI: 1.001–1.008; *p* = 0.006), corresponding to a 4.6% increase in risk per 10 µg/m^3^ increase above the WHO guideline. No association was observed for PM_2.5_. Mean temperature showed a weak inverse association with mortality (RR = 0.982 per 1 °C; 95% CI: 0.964–1.001), although this did not reach statistical significance.

#### 3.3.3. Respiratory System Mortality (RSD)

For respiratory system mortality, a pronounced seasonal pattern was observed, with substantially higher mortality in winter months and markedly lower mortality during the warmer months. Mortality was highest in January (RR = 1.283; 95% CI: 1.228–1.341) and February (RR = 1.154; 95% CI: 1.092–1.221), while the largest reductions were observed from May to October, particularly in September (RR = 0.639; 95% CI: 0.586–0.696) and October (RR = 0.669; 95% CI: 0.625–0.716). No significant difference was observed for March.

Key model estimates for respiratory system mortality are presented in Table 5.

Substantial demographic differences were observed, with markedly higher mortality among individuals aged ≥65 years compared to those aged 0–64 (RR = 101.154; 95% CI: 100.863–101.446; *p* < 0.001), and higher mortality among males compared to females (RR = 2.753; 95% CI: 2.750–2.756; *p* < 0.001). However, a significant interaction between sex and age group (RR = 0.512; 95% CI: 0.511–0.512; *p* < 0.001) indicated that the combined effect of male sex and older age was less than multiplicative, suggesting attenuation of the joint effect when both factors were present. This pattern is further illustrated in Table S11 (Supplementary Material).

Regarding environmental exposures, NO_2_ excess was positively associated with respiratory mortality (RR = 1.014 per 1 µg/m^3^; 95% CI: 1.009–1.019; *p* < 0.001), corresponding to an approximate 14.9% increase in risk per 10 µg/m^3^ increase above the WHO guideline. No evidence of association was observed for PM_2.5_ or mean temperature.

### 3.4. Sensitivity Analysis for the COVID-19 Pandemic Period

Sensitivity analyses including an indicator for the COVID-19 pandemic period (2020–2022) showed some outcome-specific differences relative to the main models (Supplementary Tables S12–S14). For circulatory mortality, the association with NO_2_ excess remained statistically significant and essentially unchanged (RR 1.004, 95% CI 1.001–1.006; *p* = 0.002). For respiratory mortality, the NO_2_ association was attenuated but remained statistically significant (RR 1.009, 95% CI 1.005–1.013; *p* < 0.001), compared with RR 1.014 (95% CI 1.009–1.019; *p* < 0.001) in the main analysis. In contrast, for malignant tumour mortality, the associations with NO_2_ excess and the NO_2_ × PM_2.5_ interaction were attenuated and no longer statistically significant after adjustment for the pandemic period. Demographic associations remained essentially unchanged across all three outcomes. The pandemic-period indicator was associated with lower malignant tumour mortality (RR 0.962, 95% CI 0.931–0.993; *p* = 0.018) and respiratory mortality (RR 0.875, 95% CI 0.835–0.918; *p* < 0.001), but not with circulatory mortality (RR 0.977, 95% CI 0.936–1.021; *p* = 0.302).

## 4. Discussion

This study shows that associations between air pollution, temperature, and mortality differ across causes of death, while mortality risks vary substantially across sex and age groups after accounting for environmental exposure conditions. Respiratory mortality showed the strongest association with NO_2_, the most pronounced seasonal pattern, and marked demographic differences. Circulatory mortality showed a significant association with NO_2_, with weaker evidence of an association with temperature and a significant interaction between sex and age group. Malignant tumour mortality exhibited weaker environmental associations overall and showed evidence of pollutant interaction in the main analysis, although these findings were less robust in sensitivity analyses. Together, these findings indicate that cause-specific mortality risks are shaped by both environmental exposure patterns and demographic structure, supporting the value of integrated models that account for co-exposure and sex–age differences in mortality risk.

The positive association between NO_2_ and circulatory mortality is consistent with previous epidemiological evidence linking traffic-related air pollution to cardiovascular risk, including meta-analyses reporting increases of 5–10% in cardiovascular mortality per 10 µg/m^3^ increase in NO_2_ [28,29]. However, the exposure metric used in this study differs from that adopted in most conventional epidemiological analyses, as it focuses on concentrations exceeding the WHO 2021 air quality guideline value rather than modelling the entire concentration range. Consequently, the estimated effects represent excess risk beyond recommended thresholds and are not directly comparable to conventional concentration–response estimates reported in the literature, although the direction and overall magnitude of the observed associations remain broadly consistent with previous evidence. The weak inverse association observed between temperature and circulatory mortality is also consistent with evidence that cold exposure may increase cardiovascular risk through vasoconstriction, elevated blood pressure, haemoconcentration, and pro-thrombotic responses [30].

The demographic patterns observed for circulatory mortality were particularly notable. Among individuals aged 0–64 years, male sex was associated with an almost three-fold higher risk of circulatory mortality compared with females (RR = 2.893; 95% CI: 2.888–2.898). However, this sex difference was largely attenuated among older adults, as shown by the negative interaction between male sex and age ≥65 years (RR for interaction = 0.344; 95% CI: 0.343–0.344). On a multiplicative scale, the expected RR for males aged ≥65 years would have been approximately 228 in the absence of interaction, whereas the model-based RR was 78.209, similar to that observed among females aged ≥65 years (RR = 78.693). These findings indicate that the male excess risk observed in younger adults was substantially reduced at older ages, where cardiovascular mortality risk was high in both sexes. This pattern may reflect the sharp increase in cardiovascular risk among post-menopausal women, together with the accumulation of cardiometabolic vulnerability and greater susceptibility to pollution-related oxidative stress, systemic inflammation, endothelial dysfunction, and atherosclerotic processes in older adults [12,31,32].

Respiratory mortality showed the strongest environmental association with NO_2_, with a 1.4% increase in risk per 1 µg/m^3^ increase above the WHO guideline value (RR = 1.014; 95% CI: 1.009–1.019), corresponding to an approximate 15% increase per 10 µg/m^3^. This finding is biologically plausible given the role of NO_2_ in airway inflammation, oxidative stress, and exacerbation of chronic respiratory disease [28,29,33]. These findings are consistent with previous analyses conducted in Portugal, particularly in the Lisbon and Porto metropolitan areas, where stronger associations with cardiorespiratory mortality have been reported [9]. Respiratory mortality also showed a pronounced seasonal pattern, with higher risks in winter months, particularly January (RR = 1.283; 95% CI: 1.228–1.341), and substantially lower risks in September and October relative to December. This winter excess is consistent with previous evidence and likely reflects the combined effects of colder temperatures, seasonal variation in pollutant concentrations, and increased circulation of respiratory infections [9].

Respiratory mortality also displayed the strongest demographic gradient among the causes of death analysed. Compared with females aged 0–64 years, males aged 0–64 years had an almost three-fold higher risk (RR = 2.753; 95% CI: 2.750–2.756), while females aged ≥65 years had a more than 100-fold higher risk (RR = 101.154; 95% CI: 100.863–101.446). The highest predicted risk was observed among males aged ≥65 years (RR = 142.610; 95% CI: 142.200–143.020). Although the sex–age interaction was submultiplicative (RR for interaction = 0.512; 95% CI: 0.511–0.512), the combined risk in older men remained markedly elevated. These locally derived, demographically stratified estimates help address the need for more granular risk assessment and are consistent with evidence that respiratory diseases are especially sensitive to age-related declines in pulmonary reserve, immunosenescence, chronic inflammation, and cumulative lifetime exposure to environmental pollutants [31,33]. The stronger NO_2_ effect observed for respiratory mortality further supports the relevance of traffic-related combustion emissions as an important determinant of respiratory vulnerability in ageing populations.

For malignant tumour mortality, environmental associations were weaker, consistent with the longer latency and multifactorial nature of cancer [3,34]. Nevertheless, interactions between pollutants together with distinct demographic patterns were observed, suggesting that susceptibility to environmental exposures may vary across causes of death. Although both NO_2_ and PM_2.5_ are established carcinogenic pollutants associated with oxidative stress and systemic inflammation [35], disentangling their independent contributions remains difficult because of their shared traffic-related sources and resulting collinearity [33,35]. The attenuation of the NO_2_ effect observed at higher PM_2.5_ levels is therefore consistent with previous multipollutant studies in which associations with NO_2_ are reduced after adjustment for PM_2.5_, potentially reflecting the influence of correlated exposures [33].

The demographic interaction observed for malignant tumour mortality revealed a distinct risk pattern. Compared with females aged 0–64 years, males aged 0–64 years had higher mortality risk (RR = 1.728; 95% CI: 1.727–1.729), while females aged ≥65 years had an approximately ten-fold higher risk (RR = 10.451; 95% CI: 10.441–10.461). The highest predicted risk was observed among males aged ≥65 years (RR = 20.166; 95% CI: 20.144–20.189). In contrast to the submultiplicative patterns observed for circulatory and respiratory mortality, the interaction between male sex and age ≥65 years was greater than multiplicative (RR for interaction ≈ 1.117), indicating an amplification of the combined sex–age effect. These estimates provide more granular evidence across population subgroups and are compatible with the cumulative nature of carcinogenic exposure, historical differences in smoking prevalence and occupational exposure, and sex-related differences in cancer susceptibility and survival [3,34]. Although the environmental associations were weaker than those observed for other outcomes, the pollutant interaction observed in the main model suggests that co-exposure structures may warrant further investigation in relation to long-term carcinogenic processes.

The sensitivity analysis incorporating the COVID-19 pandemic period provided additional context for interpreting these findings over 2010–2022. Adjustment for the pandemic-period indicator had little influence on the NO_2_ association with circulatory mortality, while the association with respiratory mortality was attenuated but remained statistically significant. These findings support the robustness of the principal NO_2_ associations with circulatory and respiratory mortality to broad temporal changes occurring during 2020–2022. In contrast, the weaker NO_2_ association and the NO_2_ × PM_2.5_ interaction observed for malignant tumour mortality were attenuated and no longer statistically significant after adjustment for the pandemic period, indicating greater sensitivity of these estimates to pandemic-related temporal changes. Demographic associations remained essentially unchanged across all three outcomes. The pandemic-period indicator itself should not be interpreted as estimating a direct effect of COVID-19, as it captures multiple concurrent changes during 2020–2022, including changes in mortality patterns, healthcare use, population behaviour, mobility, and environmental exposures.

The stronger and more consistent associations observed for NO_2_ compared with PM_2.5_ across the three mortality outcomes may reflect differences in pollutant sources, monitoring coverage, and exposure uncertainty. NO_2_ is closely linked to traffic-related combustion and is typically measured in urban areas where population density and exposure potential are highest, whereas PM_2.5_ reflects a more heterogeneous mixture of sources, including biomass burning, regional transport, secondary aerosol formation, and natural dust events. Differences in source heterogeneity, monitoring coverage, and exposure uncertainty may therefore reduce the ability of monthly aggregated PM_2.5_ indicators to capture health-relevant exposure contrasts [36]. In addition, relatively few monitoring stations in Portugal provide long and complete PM_2.5_ time series, requiring the estimation of PM_2.5_ concentrations from PM_10_ measurements as described in Section 2.1.2. Although this approach enabled the construction of consistent national exposure series, it may also have attenuated associations with PM_2.5_ through additional exposure misclassification. Therefore, the absence of statistically significant PM_2.5_ associations should not be interpreted as evidence of no health effect, but may partly reflect exposure uncertainty and co-exposure structure.

The present findings can also be interpreted in light of previous work conducted in Portugal using the World Health Organization AirQ+ tool, including our own earlier study [21]. While AirQ+ is well suited to estimating attributable mortality using predefined concentration–response functions, recent reviews have shown that many AirQ+ applications rely on externally derived relative risks and limited demographic stratification [37]. The present approach provides complementary evidence by estimating context-specific associations from observed Portuguese data and incorporating sex–age interaction structures and co-exposure patterns. Thus, while burden-of-disease frameworks remain essential for policy evaluation and international comparability, flexible epidemiological models can provide additional insight into how risks vary across exposure conditions, causes of death, and population subgroups.

This study has several strengths, including the use of Generalised Estimating Equations to account for temporal correlation, the focus on cause-specific mortality, and the systematic evaluation of interaction terms and correlation structures using QIC. The explicit modelling of sex- and age-specific interaction structures represents an additional methodological strength, as recent reviews of environmental burden-of-disease assessments, including AirQ+ studies, have identified insufficient demographic stratification as a frequent limitation [37]. The use of pollutant concentrations exceeding WHO guideline values also provides a policy-oriented framework for evaluating excess risk under real-world exposure conditions.

However, several limitations should also be considered. Because the study focuses on national-level associations and uses demographic strata with relatively low monthly counts, exposure and mortality were aggregated to monthly resolution. The resulting relative risks represent associations with monthly average environmental conditions rather than acute effects driven by daily fluctuations. In addition, the nationwide design and aggregated exposure and mortality series did not allow the assessment of geographic heterogeneity in exposure–mortality associations, including potential regional differences in pollutant sources, population vulnerability, healthcare access, or socio-economic conditions. Exposure assessment relied on ambient concentrations and may therefore introduce non-differential exposure misclassification [28,30,33]. Exposure uncertainty may have been greater for PM_2.5_, as concentrations at stations without direct PM_2.5_ measurements were estimated from PM_10_ using the empirically derived conversion factor described in Section 2.1.2, potentially introducing additional exposure misclassification. Another limitation is that malignant tumours were analysed as an aggregated outcome (ICD-10: C00–C97) because tumour-specific time series data were not available. This prevented site-specific analyses or restriction to cancers occurring in both sexes and requires caution when interpreting sex-related differences, which may partly reflect differences in the distribution of tumour types between males and females rather than sex-specific susceptibility to environmental exposures. Nevertheless, the use of an aggregated malignant tumour outcome is supported by evidence that air pollution may contribute to carcinogenesis through mechanisms such as oxidative stress, chronic inflammation, genotoxicity, and DNA damage that are relevant across multiple tumour types, while recent evidence also indicates associations between long-term air pollution exposure and cancer at multiple sites [38]. Future studies using disaggregated tumour-specific data could examine these associations more specifically. Residual confounding related to seasonal infections, humidity, or socio-economic conditions cannot be excluded, although the inclusion of calendar month and temperature likely captured part of this temporal variability [9,30,33]. Given the observational design of the study, the associations identified between environmental exposures and mortality cannot be interpreted as causal relationships. The estimated RRs should therefore be understood as measures of association rather than as evidence of causal effects. Finally, the interpretation of interactions between pollutants and temperature remains constrained by collinearity and the complexity of environmental mixtures, reflecting a broader challenge in environmental epidemiology rather than a limitation specific to this study [3,28,33].

## 5. Conclusions

This study shows that associations between air pollution, temperature, and mortality differ by cause of death, and that mortality risks vary substantially across sex and age groups after adjustment for air pollution, temperature, and seasonality. Respiratory mortality emerged as the cause of death most strongly associated with NO_2_, with pronounced seasonal variation and particularly high risks among older adults. Circulatory mortality was also associated with NO_2_ and showed strong age-related patterns, whereas malignant tumour mortality exhibited weaker environmental associations and more complex patterns, including pollutant and sex–age interactions, although the pollutant-related findings were less robust in sensitivity analyses.

These findings highlight the importance of considering environmental exposures together with demographic differences when assessing health risks. From a prevention perspective, these findings also distinguish non-modifiable susceptibility factors, such as age and sex, from potentially modifiable environmental exposures. While demographic characteristics cannot be altered, exposure to ambient air pollution can be reduced through interventions targeting pollutant emissions and population exposure. The identification of more susceptible demographic groups may therefore help prioritise protection where vulnerability is greatest, while preventive action should focus primarily on reducing the environmental exposures that contribute to risk.

By focusing on pollutant concentrations exceeding WHO guideline values, this study provides evidence of associations with mortality under these exposure conditions, offering a perspective closely aligned with regulatory and public health decision-making. At the population level, the findings support continued measures to reduce NO_2_ and particulate matter concentrations, particularly through emission reductions in areas affected by traffic and other major combustion sources. At the individual level, public-health guidance may complement these structural measures by encouraging susceptible individuals, particularly older adults, to use local air-quality information and limit prolonged exposure in highly polluted outdoor environments during periods of elevated pollution. However, because monitoring stations were used to characterise ambient pollution rather than individual exposure, their locations should not be interpreted as identifying localised areas of individual health risk.

Overall, the results suggest that approaches assuming uniform and independent effects of environmental exposures may overlook important variations in risk across exposure conditions, causes of death, and population groups. By incorporating sex–age interaction structures, co-exposure patterns, temperature, and exposure exceedance metrics within a unified analytical framework, this study addresses key gaps related to demographic stratification, multipollutant exposure, environmental context, and locally derived risk estimation. In doing so, it provides demographically stratified estimates of mortality risk based on observed Portuguese data. These findings complement standardised burden-of-disease approaches and support the development of environmental health assessment frameworks capable of incorporating interaction effects and demographic heterogeneity into future health impact assessments.

## Figures and Tables

**Table 1:** Descriptive statistics of mortality, air pollutants, and temperature in Portugal, 2010–2022.

Variable	Mean	SD	P_25_	Median	P_75_
**Malignant tumours (MNs)**					
Male, 0–64 years	357.7	26.0	341.3	355.5	374.8
Male, ≥65 years	969.8	85.4	907.3	971.5	1025.8
Female, 0–64 years	215.0	17.0	202.0	214.0	225.8
Female, ≥65 years	692.8	59.4	646.0	695.5	735.8
**Circulatory system diseases (CVD)**					
Male, 0–64 years	165.6	25.1	149.0	163.5	178.8
Male, ≥65 years	1043.0	191.1	893.8	996.0	1155.3
Female, 0–64 years	59.6	11.8	52.0	58.0	66.0
Female, ≥65 years	1447.7	256.2	1253.8	1394.5	1582.0
**Respiratory system diseases (RSD)**					
Male, 0–64 years	41.1	15.3	30.0	37.0	49.8
Male, ≥65 years	490.7	144.9	393.3	444.0	566.8
Female, 0–64 years	15.5	6.9	10.3	14.0	19.0
Female, ≥65 years	483.2	162.9	374.0	425.0	543.0
**Air pollutants**					
NO_2_ (µg/m^3^)	18.0	5.5	13.9	17.0	22.0
PM_2.5_ (µg/m^3^)	8.9	2.3	7.3	8.4	10.2
**Temperature**					
Mean temperature (T_mean_, °C)	16.1	3.5	13.1	15.9	19.2

Values are presented as mean, standard deviation (SD), and percentiles (P_25_, median, P_75_) of monthly values over the study period (2010–2022). Mortality is stratified by cause of death, sex, and age group (0–64 and ≥65 years).

**Table 2:** Summary of selected models for each mortality outcome (Portugal, 2010–2022).

Outcome	Selected Model	Interaction Terms	Correlation Structure	QIC	QICc
Malignant tumours (MNs)	M2	Sex × AgeGroup; NO_2__Excess × PM_2.5__Excess	AR(1)	1019.0	997.9
Circulatory system diseases (CVD)	M1	Sex × AgeGroup	AR(1)	711.9	691.2
Respiratory system diseases (RSD)	M1	Sex × AgeGroup	AR(1)	657.6	680.9

Selected models were identified based on the lowest quasi-likelihood under the independence model criterion (QIC). QICc denotes the corrected QIC. All models included main effects for calendar month, sex, age group, NO_2__Excess, PM_2.5__Excess, and mean temperature (T_mean_). Detailed model selection results are presented in Tables S2–S4 (Supplementary Material).

**Table 3:** Key results from the selected model for malignant tumour mortality (Portugal, 2010–2022).

Variable	RR	95% CI	*p*-Value
**Demographic variables**			
Male (vs. Female)	1.728	1.727–1.729	<0.001
Age ≥65 (vs. 0–64)	10.451	10.441–10.461	<0.001
**Environmental variables**			
NO_2__Excess	1.003	1.001–1.006	0.019
PM_2.5__Excess	1.001	0.997–1.005	0.579
T_mean_ (per 1 °C)	0.997	0.989–1.004	0.406
**Interaction terms**			
Male × Age ≥ 65	1.1169	1.1167–1.1171	<0.001
NO_2__Excess × PM_2.5__Excess	0.9998	0.9997–0.9999	0.003

Relative risks (RR) were obtained by exponentiating model coefficients. Estimates for NO_2__Excess and PM_2.5__Excess represent the effect per 1 µg/m^3^ increase above the 2021 World Health Organization (WHO) air quality guideline values. Interaction terms represent departures from multiplicative effects. All estimates are adjusted for calendar month and other covariates included in the model. Detailed parameter estimates are provided in Table S5 (Supplementary Material).

**Table 4:** Key results from the selected model for circulatory system mortality (Portugal, 2010–2022).

Variable	RR	95% CI	*p*-Value
**Demographic variables**			
Male (vs. Female)	2.893	2.888–2.898	<0.001
Age ≥65 (vs. 0–64)	78.693	78.376–79.011	<0.001
**Environmental variables**			
NO_2__Excess	1.005	1.001–1.008	0.006
PM_2.5__Excess	1.001	0.993–1.008	0.869
T_mean_ (per 1 °C)	0.982	0.964–1.001	0.067
**Interaction terms**			
Male × Age ≥ 65	0.344	0.343–0.344	<0.001

Relative risks (RR) were obtained by exponentiating model coefficients. Estimates for NO_2__Excess and PM_2.5__Excess represent the effect per 1 µg/m^3^ increase above the 2021 World Health Organization (WHO) air quality guideline values. Interaction terms represent departures from multiplicative effects. All estimates are adjusted for calendar month and other covariates included in the model. Detailed parameter estimates are provided in Table S8 (Supplementary Material).

**Table 5:** Key results from the selected model for respiratory system mortality (Portugal, 2010–2022).

Variable	RR	95% CI	*p*-Value
**Demographic variables**			
Male (vs. Female)	2.753	2.750–2.756	<0.001
Age ≥65 (vs. 0–64)	101.154	100.863–101.446	<0.001
**Environmental variables**			
NO_2__Excess	1.014	1.009–1.019	<0.001
PM_2.5__Excess	1.000	0.992–1.007	0.933
T_mean_ (per 1 °C)	1.001	0.993–1.008	0.886
**Interaction terms**			
Male × Age ≥ 65	0.512	0.511–0.512	<0.001

Relative risks (RR) were obtained by exponentiating model coefficients. Estimates for NO_2__Excess and PM_2.5__Excess represent the effect per 1 µg/m^3^ increase above the 2021 World Health Organization (WHO) air quality guideline values. Interaction terms represent departures from multiplicative effects. All estimates are adjusted for calendar month and other covariates included in the model. Detailed parameter estimates are provided in Table S10 (Supplementary Material).

## Data Availability

The original contributions presented in this study are included in the article/Supplementary Material. Further inquiries can be directed to the corresponding author.

## Supplementary Materials

The following supporting information can be downloaded at: https://www.mdpi.com/article/10.3390/jox16050172/s1, Table S1: Annual number of monitoring stations contributing NO_2_ and PM_2.5_ data, including directly measured and PM_10_-derived PM_2.5_ concentrations (Portugal, 2010–2022); Table S2: Model selection for malignant tumour mortality (Portugal, 2010–2022); Table S3: Model selection for diseases of the circulatory system mortality (Portugal, 2010–2022); Table S4: Model selection for diseases of the respiratory system mortality (Portugal, 2010–2022); Table S5: Results from selected model for malignant tumour mortality (Portugal, 2010–2022); Table S6: Predicted relative risks of malignant tumour mortality by sex and age group (Portugal, 2010–2022); Table S7: Conditional effects of NO_2_ on malignant tumour mortality at different PM_2.5_ levels (Portugal, 2010–2022); Table S8: Results from selected model for diseases of the circulatory system mortality (Portugal, 2010–2022); Table S9: Predicted relative risks of circulatory system mortality by sex and age group (Portugal, 2010–2022); Table S10: Results from selected model for diseases of the respiratory system mortality (Portugal, 2010–2022); Table S11: Predicted relative risks of respiratory system mortality by sex and age group (Portugal, 2010–2022); Table S12: Sensitivity analysis of the selected model for malignant tumour mortality including the COVID-19 period indicator (Portugal, 2010–2022); Table S13: Sensitivity analysis of the selected model for diseases of the circulatory system mortality including the COVID-19 period indicator (Portugal, 2010–2022); Table S14: Sensitivity analysis of the selected model for diseases of the respiratory system mortality including the COVID-19 period indicator (Portugal, 2010–2022); Figure S1: Interaction between NO_2_ and PM_2.5_ on malignant tumour mortality (Portugal, 2010–2022).

## Abbreviations

The following abbreviations are used in this manuscript:

APA	Portuguese Environment Agency
AQG	Air Quality Guidelines
AR(1)	First-order autoregressive correlation structure
COPD	Chronic obstructive pulmonary disease
CVD	Circulatory System Diseases
EEA	European Environment Agency
GBD	Global Burden of Disease
GEE	Generalised Estimating Equations
ICD	International Classification of Diseases
INE	Statistics Portugal
IPMA	Portuguese Institute for Sea and Atmosphere
RR	Relative Risk
MNs	Malignant Neoplasms
NO_2_	Nitrogen dioxide
NUTS	Nomenclature of Territorial Units for Statistics
PM*_x_*	Particulate matter with an aerodynamic diameter ≤ *x* µm (*x* = 10, coarse fraction; *x* = 2.5, fine fraction)
QIC	Quasi-likelihood under Independence Model Criterion
QICc	Corrected Quasi-likelihood under Independence Model Criterion
QualAr	Portuguese Air Quality Monitoring Network managed by APA
RSD	Respiratory System Diseases
Tair2m	Air temperature at 2 m above ground level
T_mean_	Monthly mean air temperature
UN	United Nations
WHO	World Health Organisation
