# Supplementary material for: Air Pollution, Temperature, and Demographic Vulnerability as Determinants of Cause-Specific Mortality in Portugal
Source: J Xenobiot. 2026 Sep 12;16(5):172. doi: 10.3390/jox16050172 (PMC13608210; doi:10.3390/jox16050172)
Supplement: Supplementary file 1 [file jox-16-00172-s001.zip › jox-4490073-supplementary.pdf]

Supplementary Materials: Air pollution, temperature, and demographic vulnerability as determinants of cause-specific mortality in Portugal

João Simões, Alexandra Bernardo, Luísa Lima Gonçalves and José Brito

Contents

Supplementary Tables ***2***

Table S1. Annual number of monitoring stations contributing NO_2 and $PM_{2.5}$ data, including directly measured and PM_{10}-derived $PM_{2.5}$ concentrations (Portugal, 2010–2022) 2

Table S2. Model selection for malignant tumour mortality (Portugal, 2010–2022) 3

Table S3. Model selection for diseases of the circulatory system mortality (Portugal, 2010–2022) 3

Table S4. Model selection for diseases of the respiratory system mortality (Portugal, 2010–2022) 3

Table S5. Results from selected model for malignant tumour mortality (Portugal, 2010–2022) 4

Table S6. Predicted relative risks of malignant tumour mortality by sex and age group (Portugal, 2010–2022) 4

Table S7. Conditional effects of NO_2 on malignant tumour mortality at different $PM_{2.5}$ levels (Portugal, 2010–2022) 5

Table S8. Results from selected model for diseases of the circulatory system mortality (Portugal, 2010–2022) 5

Table S9. Predicted relative risks of circulatory system mortality by sex and age group (Portugal, 2010–2022) 6

Table S10. Results from selected model for diseases of the respiratory system mortality (Portugal, 2010–2022) 6

Table S11. Predicted relative risks of respiratory system mortality by sex and age group (Portugal, 2010–2022) 7

Table S12. Sensitivity analysis of the selected model for malignant tumour mortality including the COVID-19 period indicator (Portugal, 2010–2022) 8

Table S13. Sensitivity analysis of the selected model for diseases of the circulatory system mortality including the COVID-19 period indicator (Portugal, 2010–2022) 8

Table S14. Sensitivity analysis of the selected model for diseases of the respiratory system mortality including the COVID-19 period indicator (Portugal, 2010–2022) 8

Supplementary Figures ***9***

Figure S1. Interaction between NO_2 and $PM_{2.5}$ on malignant tumour mortality (Portugal, 2010–2022) 9

Supplementary Tables

Table S1. Annual number of monitoring stations contributing NO_2 and $PM_{2.5}$ data, including directly measured and PM_{10}-derived $PM_{2.5}$ concentrations (Portugal, 2010–2022).

Year	Total NO_2, n	Direct $PM_{2.5}$, n (%)	PM_{10}-derived $PM_{2.5}$, n (%)	Total $PM_{2.5}$, n
2010	58	17 (31.5)	37 (68.5)	54
2011	51	16 (32.0)	34 (68.0)	50
2012	48	14 (30.4)	32 (69.6)	46
2013	41	12 (29.3)	29 (70.7)	41
2014	43	12 (31.6)	26 (68.4)	38
2015	48	15 (31.9)	32 (68.1)	47
2016	41	15 (33.3)	30 (66.7)	45
2017	35	14 (31.8)	30 (68.2)	44
2018	45	12 (24.5)	37 (75.5)	49
2019	46	17 (37.0)	29 (63.0)	46
2020	45	18 (43.9)	23 (56.1)	41
2021	45	20 (48.8)	21 (51.2)	41
2022	56	21 (50.0)	21 (50.0)	42

Note: Percentages for direct and PM_{10}-derived $PM_{2.5}$ were calculated relative to the total number of monitoring stations contributing $PM_{2.5}$ data in each year. "Direct $PM_{2.5}$" refers to stations providing valid direct $PM_{2.5}$ measurements, whereas "PM_{10}-derived $PM_{2.5}$" refers to stations providing valid PM_{10} measurements from which $PM_{2.5}$ concentrations were estimated using the empirically derived conversion factor of 0.45. PM_{10} was not analysed as a separate exposure variable. Counts refer to monitoring stations contributing valid data within each year and not to individual hourly or daily observations.

Table S2. Model selection for malignant tumour mortality (Portugal, 2010–2022)

Model	Interaction	Correlation Structure	QIC	QICc	Selected
M0	None	Independent	1412.8	1151.8	
M0	None	AR(1)	1411.7	1159.1	
M1	Sex × AgeGroup	Independent	1022.6	993.0	
M1	Sex × AgeGroup	AR(1)	1019.1	996.5	
M2	Sex × AgeGroup; NO_2_Excess × $PM_{2.5}$_Excess	Independent	1023.5	994.1	
M2	Sex × AgeGroup; NO_2_Excess × $PM_{2.5}$_Excess	AR(1)	1019.0	997.9	✓
M3	Sex × AgeGroup; NO_2_Excess × T_{mean}	Independent	1024.0	995.0	
M3	Sex × AgeGroup; NO_2_Excess × T_{mean}	AR(1)	1020.5	999.2	
M4	Sex × AgeGroup; $PM_{2.5}$_Excess × T_{mean}	Independent	1024.1	994.5	
M4	Sex × AgeGroup; $PM_{2.5}$_Excess × T_{mean}	AR(1)	1019.9	998.1	

Note: QIC: Quasi-likelihood under the independence model criterion; QICc: corrected QIC. Lower values indicate better model fit. M0 included month, sex, age group, NO_2_Excess, $PM_{2.5}$_Excess, and mean temperature (T_{mean}). The model with the lowest QIC was selected.

Table S3. Model selection for diseases of the circulatory system mortality (Portugal, 2010–2022)

Model	Interaction	Correlation Structure	QIC	QICc	Selected
M0	None	Independent	1412.3	669.9	
M0	None	AR(1)	1372.7	675.0	
M1	Sex × AgeGroup	Independent	750.2	689.1	
M1	Sex × AgeGroup	AR(1)	711.9	691.2	✓
M2	Sex × AgeGroup; NO_2_Excess × $PM_{2.5}$_Excess	Independent	751.6	690.8	
M2	Sex × AgeGroup; NO_2_Excess × $PM_{2.5}$_Excess	AR(1)	714.2	695.9	
M3	Sex × AgeGroup; NO_2_Excess × T_{mean}	Independent	751.5	690.3	
M3	Sex × AgeGroup; NO_2_Excess × T_{mean}	AR(1)	714.1	694.7	
M4	Sex × AgeGroup; $PM_{2.5}$_Excess × T_{mean}	Independent	753.6	691.0	
M4	Sex × AgeGroup; $PM_{2.5}$_Excess × T_{mean}	AR(1)	714.6	694.3	

Note: See Table S2.

Table S4. Model selection for diseases of the respiratory system mortality (Portugal, 2010–2022)

Model	Interaction	Correlation Structure	QIC	QICc	Selected
M0	None	Independent	1048.2	714.5	
M0	None	AR(1)	1039.3	717.1	
M1	Sex × AgeGroup	Independent	661.1	678.0	
M1	Sex × AgeGroup	AR(1)	657.6	680.9	✓
M2	Sex × AgeGroup; NO_2_Excess × $PM_{2.5}$_Excess	Independent	662.8	680.7	
M2	Sex × AgeGroup; NO_2_Excess × $PM_{2.5}$_Excess	AR(1)	659.2	683.8	
M3	Sex × AgeGroup; NO_2_Excess × T_{mean}	Independent	661.0	679.7	
M3	Sex × AgeGroup; NO_2_Excess × T_{mean}	AR(1)	658.8	684.1	
M4	Sex × AgeGroup; $PM_{2.5}$_Excess × T_{mean}	Independent	661.5	680.6	
M4	Sex × AgeGroup; $PM_{2.5}$_Excess × T_{mean}	AR(1)	657.9	683.3	

Note: See Table S2.

Table S5. Results from selected model for malignant tumour mortality (Portugal, 2010–2022)

Variable	Coefficient	RR	95% CI	*p*-value
**Month**				
January	0.021	1.022	1.003 – 1.040	0.020
February	-0.107	0.899	0.888 – 0.910	<0.001
March	-0.043	0.958	0.949 – 0.967	<0.001
April	-0.078	0.925	0.913 – 0.938	<0.001
May	-0.040	0.961	0.934 – 0.988	0.006
June	-0.055	0.947	0.897 – 1.000	0.049
July	-0.015	0.985	0.915 – 1.060	0.687
August	-0.011	0.989	0.922 – 1.061	0.752
September	-0.053	0.949	0.904 – 0.996	0.033
October	-0.022	0.978	0.948 – 1.010	0.177
November	-0.046	0.955	0.940 – 0.969	<0.001
December	Reference	—	—	—
**Demographic variables**				
Male (vs Female)	0.547	1.728	1.727 – 1.729	<0.001
Age ≥65 (vs 0–64)	2.347	10.451	10.441 – 10.461	<0.001
**Environmental variables**				
NO_2_Excess	0.003	1.003	1.001 – 1.006	0.019
$PM_{2.5}$_Excess	0.001	1.001	0.997 – 1.005	0.579
T_mean (per 1°C)	-0.003	0.997	0.989 – 1.004	0.406
**Interaction terms**				
Male × Age ≥65	0.1105	1.1169	1.1167 – 1.1171	<0.001
NO_2_Excess × $PM_{2.5}$_Excess	-0.0002	0.9998	0.9997 – 0.9999	0.003

Coefficients for NO_2_Excess and $PM_{2.5}$_Excess represent the effect per 1 μg/m^3 increase above the 2021 WHO air quality guideline (AQG) values. The interaction terms indicate how the effect of each pollutant changes with increasing levels of the other and the combined effect of sex and age group.

Table S6. Predicted relative risks of malignant tumour mortality by sex and age group (Portugal, 2010–2022)

Group	RR	95% CI
Female 0–64	1.000	—
Male 0–64	1,728	1.727 – 1.729
Female ≥65	10,451	10.441 – 10.461
Male ≥65	20,166	20.144 – 20.189

Relative risks (RR) were derived from the interaction model (M2), using females aged 0–64 years as the reference group. Estimates for each group were obtained by combining the corresponding regression coefficients. Standard errors and 95% confidence intervals were calculated using the full variance–covariance matrix of the model coefficients. Estimates were computed holding NO_2 and $PM_{2.5}$ excess at 0 μg/m^3, month in December, and mean temperature at its average value.

Table S7. Conditional effects of NO_2 on malignant tumour mortality at different $PM_{2.5}$ levels (Portugal, 2010–2022)

$PM_{2.5}$_Excess (μg/m³)	β_cond (NO_2)	RR (per 10 μg/m³ NO_2)	95% CI
0	0.0034	1.034	1.005 – 1.064
1	0.0032	1.032	1.004 – 1.062
2	0.0030	1.030	1.002 – 1.060
3	0.0028	1.028	1.000 – 1.057
4	0.0026	1.027	0.999 – 1.055
5	0.0024	1.025	0.997 – 1.053
6	0.0022	1.023	0.995 – 1.051
7	0.0020	1.021	0.993 – 1.049
8	0.0019	1.019	0.991 – 1.048
9	0.0017	1.017	0.989 – 1.046
10	0.0015	1.015	0.987 – 1.044

Conditional effects were derived from the interaction model (M2) by estimating the effect of NO_2 at fixed levels of $PM_{2.5}$ excess (β_cond = β_NO_2 + β_interaction × $PM_{2.5}$_Excess). Relative risks (RR) correspond to a 10 μg/m³ increase in NO_2 above the 2021 World Health Organization (WHO) air quality guideline. Standard errors and 95% confidence intervals were calculated using the variance–covariance matrix of the model coefficients.

Table S8. Results from selected model for diseases of the circulatory system mortality (Portugal, 2010–2022)

Variable	Coefficient	RR	95% CI	*p*-value
**Month**				
January	0.084	1.088	1.060 – 1.116	<0.001
February	-0.045	0.956	0.931 – 0.982	<0.001
March	-0.034	0.967	0.913 – 1.024	0.250
April	-0.124	0.883	0.810 – 0.963	0.005
May	-0.102	0.903	0.818 – 0.996	0.042
June	-0.156	0.856	0.759 – 0.965	0.011
July	-0.091	0.913	0.789 – 1.057	0.224
August	-0.112	0.894	0.775 – 1.030	0.121
September	-0.209	0.811	0.730 – 0.901	<0.001
October	-0.168	0.845	0.785 – 0.910	<0.001
November	-0.146	0.865	0.841 – 0.889	<0.001
December	Reference	—	—	—
**Demographic variables**				
Male (vs Female)	1.062	2.893	2.888 – 2.898	<0.001
Age ≥65 (vs 0–64)	4.366	78.693	78.376 – 79.011	<0.001
**Environmental variables**				
NO_2_Excess	0.005	1.005	1.001 – 1.008	0.006
$PM_{2.5}$_Excess	0.001	1.001	0.993 – 1.008	0.869
T_mean (per 1°C)	-0.018	0.982	0.964 – 1.001	0.067
**Interaction terms**				
Male × Age ≥65	-1.068	0.344	0.343 – 0.344	<0.001

Coefficients for NO_2_Excess and $PM_{2.5}$_Excess represent the effect per 1 μg/m³ increase above the 2021 WHO air quality guideline (AQG) values. The interaction term represents the combined effect of sex and age group.

Table S9. Predicted relative risks of circulatory system mortality by sex and age group (Portugal, 2010–2022)

Group	RR	95% CI
Female 0–64	1.000	—
Male 0–64	2.893	2.888 – 2.898
Female ≥65	78.693	78.376 – 79.011
Male ≥65	78.209	77.898 – 78.521

Relative risks (RR) were derived from the interaction model (M1), using females aged 0–64 years as the reference group. Estimates for each group were obtained by combining the corresponding regression coefficients. Standard errors and 95% confidence intervals were calculated using the full variance–covariance matrix of the model coefficients. Estimates were computed holding NO_2 and $PM_{2.5}$ excess at 0 μg/m^3, month in December, and mean temperature at its average value.

Table S10. Results from selected model for diseases of the respiratory system mortality (Portugal, 2010–2022)

Variable	Coefficient	RR	95% CI	*p*-value
**Month**				
January	0.249	1.283	1.228 – 1.341	<0.001
February	0.144	1.154	1.092 – 1.221	<0.001
March	0.045	1.046	0.984 – 1.111	0.150
April	-0.166	0.847	0.765 – 0.939	0.002
May	-0.265	0.767	0.687 – 0.858	<0.001
June	-0.336	0.714	0.625 – 0.817	<0.001
July	-0.293	0.746	0.648 – 0.858	<0.001
August	-0.339	0.713	0.622 – 0.816	<0.001
September	-0.448	0.639	0.586 – 0.696	<0.001
October	-0.402	0.669	0.625 – 0.716	<0.001
November	-0.291	0.748	0.722 – 0.774	<0.001
December	Reference	—	—	—
**Demographic variables**				
Male (vs Female)	1.013	2.753	2.750 – 2.756	<0.001
Age ≥65 (vs 0–64)	4.617	101.154	100.863 – 101.446	<0.001
**Environmental variables**				
NO_2_Excess	0.014	1.014	1.009 – 1.019	<0.001
$PM_{2.5}$_Excess	-0.0003	1.000	0.992 – 1.007	0.933
T_mean (per 1°C)	0.001	1.001	0.993 – 1.008	0.886
**Interaction terms**				
Male × Age ≥65	-0.670	0.512	0.511 – 0.512	<0.001

Coefficients for NO_2_Excess and $PM_{2.5}$_Excess represent the effect per 1 μg/m^3 increase above the 2021 WHO air quality guideline (AQG) values. The interaction term represents the combined effect of sex and age group.

Table S11. Predicted relative risks of respiratory system mortality by sex and age group (Portugal, 2010–2022)

Group	RR	95% CI
Female 0–64	1.000	—
Male 0–64	2.753	2.750 – 2.756
Female ≥65	101.154	100.863 – 101.446
Male ≥65	142.610	142.200 – 143.020

Relative risks (RR) were derived from the interaction model (M1), using females aged 0–64 years as the reference group. Estimates for each group were obtained by combining the corresponding regression coefficients. Standard errors and 95% confidence intervals were calculated using the full variance–covariance matrix of the model coefficients. Estimates were computed holding NO_2 and $PM_{2.5}$ excess at 0 μg/m^3, month in December, and mean temperature at its average value.

Table S12. Sensitivity analysis of the selected model for malignant tumour mortality including the COVID-19 period indicator (Portugal, 2010–2022)

Variable	Main model RR (95% CI)	+ COVID RR (95% CI)	Main *p*	+ COVID *p*	Interpretation
Male (vs Female)	1.728 (1.727–1.729)	1.728 (1.727–1.729)	<0.001	<0.001	Essentially unchanged
Age ≥65 (vs 0–64)	10.451 (10.441–10.461)	10.456 (10.442–10.469)	<0.001	<0.001	Essentially unchanged
COVID-19 period	—	0.962 (0.931–0.993)	—	0.018	Lower mortality during 2020–2022
NO_2_Excess	1.003 (1.001–1.006)	1.000 (1.000–1.001)	0.019	0.367	Effect attenuated; no longer significant
$PM_{2.5}$_Excess	1.001 (0.997–1.005)	0.999 (0.995–1.003)	0.579	0.639	Remains non-significant
T_mean	0.997 (0.989–1.004)	1.000 (0.993–1.006)	0.406	0.951	Remains non-significant
Male × Age ≥65	1.1169 (1.1167–1.1171)	1.1168 (1.1165–1.1171)	<0.001	<0.001	Essentially unchanged
NO_2 × $PM_{2.5}$	0.9998 (0.9997–0.9999)	≈1.000	0.003	0.617	Interaction disappears after COVID adjustment

Table S13. Sensitivity analysis of the selected model for diseases of the circulatory system mortality including the COVID-19 period indicator (Portugal, 2010–2022)

Variable	Main model RR (95% CI)	+ COVID RR (95% CI)	Main *p*	+ COVID *p*	Interpretation
Male (vs Female)	2.893 (2.888–2.898)	2.894 (2.889–2.899)	<0.001	<0.001	Essentially unchanged
Age ≥65 (vs 0–64)	78.693 (78.376–79.011)	78.701 (78.366–79.037)	<0.001	<0.001	Essentially unchanged
COVID-19 period	—	0.977 (0.936–1.021)	—	0.302	No independent association
NO_2_Excess	1.005 (1.001–1.008)	1.004 (1.001–1.006)	0.006	0.002	Remains significant; essentially stable
$PM_{2.5}$_Excess	1.001 (0.993–1.008)	1.001 (0.993–1.009)	0.869	0.825	Remains non-significant
T_mean	0.982 (0.964–1.001)	0.983 (0.966–1.001)	0.067	0.065	Essentially unchanged; borderline non-significant
Male × Age ≥65	0.344 (0.343–0.344)	0.343 (0.343–0.344)	<0.001	<0.001	Essentially unchanged

Table S14. Sensitivity analysis of the selected model for diseases of the respiratory system mortality including the COVID-19 period indicator (Portugal, 2010–2022)

Variable	Main model RR (95% CI)	+ COVID RR (95% CI)	Main *p*	+ COVID *p*	Interpretation
Male (vs Female)	2.753 (2.750–2.756)	2.756 (2.754–2.759)	<0.001	<0.001	Essentially unchanged
Age ≥65 (vs 0–64)	101.154 (100.863–101.446)	101.209 (100.993–101.426)	<0.001	<0.001	Essentially unchanged
COVID-19 period	—	0.875 (0.835–0.918)	—	<0.001	Lower mortality during 2020–2022
NO_2_Excess	1.014 (1.009–1.019)	1.009 (1.005–1.013)	<0.001	<0.001	Attenuated but remains significant
$PM_{2.5}$_Excess	1.000 (0.992–1.007)	1.001 (0.994–1.008)	0.933	0.721	Remains non-significant
T_mean	1.001 (0.993–1.008)	1.006 (0.999–1.012)	0.886	0.099	Still non-significant; estimate shifts upward
Male × Age ≥65	0.512 (0.511–0.512)	0.511 (0.510–0.511)	<0.001	<0.001	Essentially unchanged

Supplementary Figures

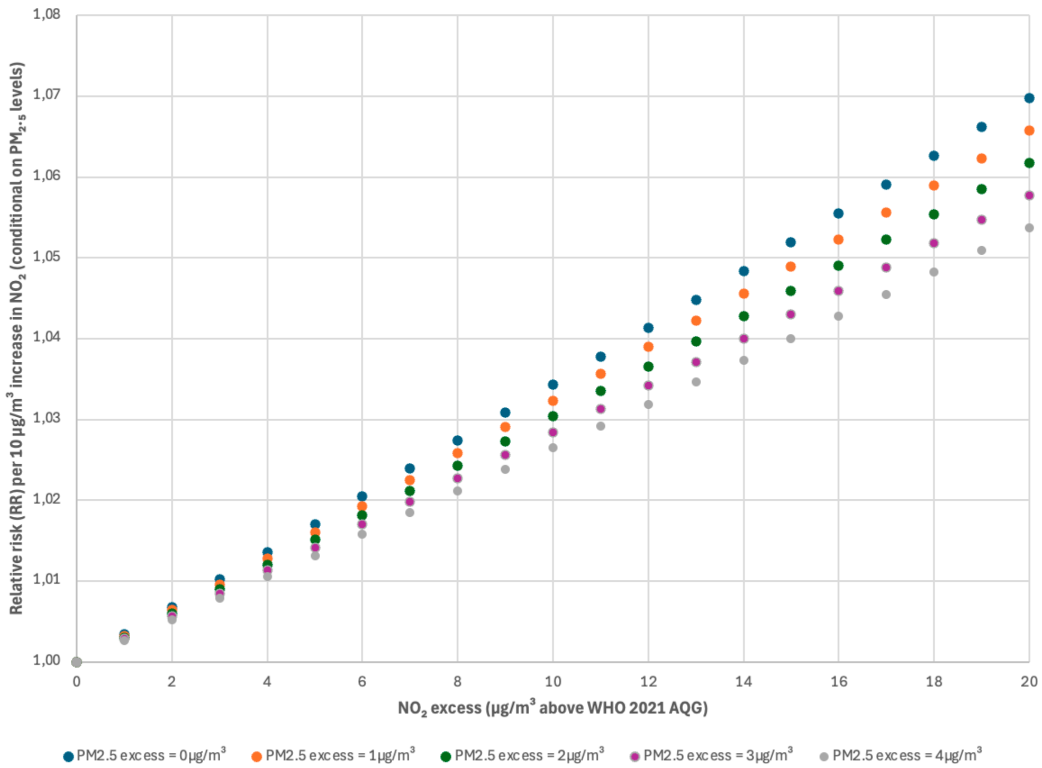

Figure S1. Interaction between NO_2 and $PM_{2.5}$ on malignant tumour mortality (Portugal, 2010–2022). Relative risks (RR) of malignant tumour mortality associated with increasing NO_2 excess levels are shown for selected fixed levels of $PM_{2.5}$ excess (0–4 μg/m^3 above WHO 2021 AQG). Each line represents the estimated RR per 1 μg/m^3 increase in NO_2, derived from the interaction model (M2). The pattern illustrates attenuation of the NO_2 effect with increasing $PM_{2.5}$ levels.
